# Estimating the rate and determinants of exclusive breastfeeding practices among rural mothers in Southern Ghana

**DOI:** 10.1186/s13006-020-0253-6

**Published:** 2020-02-07

**Authors:** Alfred Kwesi Manyeh, Alberta Amu, David Etsey Akpakli, John E. Williams, Margaret Gyapong

**Affiliations:** 1 Dodowa Health Research Centre, Dodowa, Ghana; 2 Division of Epidemiology and Biostatistics, School of Public Health, University of the Witwatersrand, Johannesburg, Parktown, South Africa; 3 University of Health and Allied Sciences, Ho, Volta Region Ghana; 4 Ghana Health Service, Accra, Ghana

**Keywords:** Exclusive breastfeeding, Child health, Demographic surveillance, Dodowa, Ghana

## Abstract

**Background:**

The health benefits of exclusive breastfeeding practices in both the short and long term accrue to breastfed infants, mothers, families and the society at large. Despite the evidence of these benefits and adoption of various World Health Organization (WHO) strategies on promotion of exclusive breastfeeding by Ghana, the increase in the rate of exclusive breastfeeding has been very slow in the country. This study aimed to estimate the rate and investigate socio-economic and demographic determinants of 6 months exclusive breastfeeding in two rural districts in Southern Ghana.

**Methods:**

Pregnancy, childbirth, breastfeeding, demographic and socioeconomic information of 1870 women who were prospectively registered by the Dodowa Health and Demographic Surveillance System and gave birth between 1 January 2011 and 31 December 2013 was extracted. The proportion of 6 months exclusive breastfeeding among the study participants was estimated and the relationship between the dependent and the independent variables were explored using logistics regression model at 95% confidence level.

**Results:**

The proportion of mothers who exclusive breastfed for 6 months in the study was 71.0%. Mothers aged 25–29 and 30 + years are 93 and 91% respectively more likely to practice 6 months exclusive breastfeeding compared to those aged < 20 years (OR 1.93, 95% CI 1.25, 2.99, OR 1.91, 95% CI 1.91, 3.08). The odds of artisan mothers practicing 6 months exclusive breastfeeding is 36% less likely compared to those unemployed (OR 0.64, 95% CI 0.43, 0.96). There is a higher chance that 45% of mothers with a household size of more than five members to practice exclusive breastfeeding compared to those with household size of less than six (OR 1.45, 95% CI 1.16, 1.81). Women in the fishing district were 85% less likely to practice 6 months exclusive breastfeeding compared to those in farming district (OR 0.15, 95% CI 0.12, 0.20).

**Conclusion:**

There is high rate of exclusive breastfeeding in the study area. Maternal age, type of occupation, household size and district of residence are determinants of 6 months exclusive breastfeeding among the study participants.

## Background

The association between child health and development outcomes with appropriate breastfeeding practices such as early initiation at birth, exclusive breastfeeding during the first 6 months of life, and breastfeeding for at least 2 years has been established [1, 2]. A baby’s diet during the first few months of life has a significant role on the composition and stability of the gut microbiome that can only be acquired after birth [3]. These bacteria make digestion of solids easier, thus, preventing gut problems and illnesses at later stage of life [3]. The World Health Organization (WHO) endorsed exclusive breastfeeding as an optimal way to feed infants [4]. Exclusive breastfeeding is defined by WHO as feeding an infant exclusively with breast milk for the first 6 months of life. During this period, the infant may be given drops of vitamins, minerals and Oral Rehydration Solution, if prescribed [4]. The WHO and UNICEF recommendations on breastfeeding includes: commencement of breastfeeding within the first hour after childbirth; exclusive breastfeeding for the first 6 months after birth; and sustained breastfeeding for 2 years or more, coupled with safe, nutritionally suitable, age appropriate, responsive complementary feeding starting in the 6 month [5].

An enhanced breastfeeding practice is known to have reduced child morbidity and mortality, improved quality of life, and enriched human capital [1, 2]. Breastfeeding is also associated with positive maternal outcomes such as reduced likelihood of breast and ovarian cancer, diabetes, and increased birth spacing [6]. In countries where child survival and growth are often endangered by infectious diseases and malnutrition, the benefits of enhanced breastfeeding practices cannot be over emphasized [7–10].

Exclusive breastfeeding practices are influenced by multiple factors. These include health, psychosocial, cultural, social, and economic factors [11, 12]. Studies have shown that the decisions regarding exclusive breastfeeding in low-income countries are influenced by education, employment, place of delivery, family pressure, and cultural values [13–15].

Other studies have shown that mixed feeding is associated with increased diarrhea and pneumonia/respiratory diseases in children [16–20]. Infants who were not exclusively breastfed have a 165% higher likelihood of suffering from diarrhea and 107% higher likelihood of pneumonia than children who were exclusively breastfed [17, 20].

Despite the evidence on benefits of exclusive breastfeeding, in 2016 only 43% of infants were exclusively breastfed globally [21]. The rate is lower (37%) in low and middle income countries [2]. Despite high child mortality and malnutrition in Sub Saharan Africa (SSA), only 36% of the infants were exclusively breastfed in 2016 [8, 21, 22].

In 2017, infant and under-5 mortality rates for Ghana were 37 and 52 deaths per 1000 live births, respectively. The neonatal mortality rate for the same period was 25 deaths per 1000 live births. At these mortality levels, one out of 19 Ghanaian children does not survive to their fifth birthday [23].

Although Ghana adopted various WHO strategies to promote exclusive breastfeeding, there has been a slow increase in the rates of exclusive breastfeeding in the country. The 2011 Ghana Multiple Indicator Cluster Survey (MICS) in 2011 reported that less than half (46%) of all infants aged 0–6 months in Ghana were exclusively breastfed [24]. This level is lower than that recommended by WHO/UNICEF.

In Ghana, 52 % of children younger than 6 months were exclusively breastfed in 2014 [25]. According to the 2014 Ghana Demographic and Health Survey (GDHS) the percentage of children aged 0–5 months who were exclusively breastfed has decreased by 17% between 2008 and 2014 [25]. The percentage of children who were bottle fed appears to have increased over the past decade. In 2003 and 2014, 11 and 16% of children under 6 months, respectively, were bottled fed [25]. The 2014 GDHS shows that the country still faces the challenges of high infant mortality of 41 deaths per 1000 live births and 19% of children were stunted ascribable to malnutrition and infections [25].

Using data from an open cohort longitudinal population, we estimated the rate of exclusive breastfeeding, examined socio-economic and demographic factors influencing exclusive breastfeeding practices in two rural districts in Southern Ghana.

## Methods

### Study area and data source

This study was conducted in the Shai-Osudoku and Ningo-Prampram districts of the Greater Accra Region of Ghana. The two districts cover a total population of 115,754 individuals living in 380 communities in 23,647 households [26]. A comprehensive description of the study districts and the operations of Dodowa Health and Demographic Surveillance System (DHDSS) can be found elsewhere [27–29]. Health service delivery in the study districts is provided by government hospitals, health centres, clinics, Community-based Health and Planning Services (CHPS) compounds/zones, missions and non-governmental health facilities [26, 27]. The secondary data was extracted from the longitudinal population-based database of the DHDSS. The extracted data was exported to STATA version 14.2 for cleaning, coding and analysis.

### Study population

All women resident in the two study districts who were registered in the DHDSS and gave birth between January 1, 2011 and December 31, 2013 were included in the study. The index baby must at least be 6 months old at the time of interview to qualify the mother to participate in the study. Women who were not registered in the DHDSS, those who delivered before 1 January 2011 or after 31 December 2013 and those whose babies were less than 6 months at the time of the interview were excluded from the study. A total of 1870 mothers were included in the study.

### Variables

#### Dependent variable

The dependent variable is breastfeeding and it was coded as 1 for 6 months exclusive breastfeeding and 0 if otherwise. Six months exclusive breastfeeding in this study is defined as feeding infants with only breast milk, without supplemental liquids or solids except for liquid medicine and vitamin or mineral supplements for the first 6 months of life [25]. This is based on UNICEF and WHO recommendation [30]. Questions on exclusive breastfeeding was administered to all women who gave birth between January 1, 2011 and December 31, 2013. The question was asked retrospectively from the time of birth to the first 6 months of life of the index child.

#### Independent variables

From the available DHDSS data, we extracted nine independent variables which includes; maternal age, educational level, marital status, parity, timing of antenatal clinic initiation, place of delivery, educational level of household head, district of residence and socio-economic status. Being “married” in this study refers to performance of a legal marriage ceremony including payment of bride price; “cohabitation” is when a man and a woman live together for the purpose of marriage without a legal marriage ceremony and without payment of bride price.

“Timing of antenatal clinic initiation” is the period during pregnancy when the expectant mother started accessing antenatal care (ANC) services. The “timing of antenatal clinic initiation” in this is grouped into three categories (first, second and third trimesters).

The socio-economic status is estimated using weights derived from principal component analysis (PCA) through household social status, ownership of assets, availability of utilities among others [26, 27]. Household socioeconomic status is a proxy measure of a household’s long term standard of living [27]. The proxies from the PCA were divided into five quintiles; poorest, poorer, middle, richer and richest [26, 27]. Selection of these variables was also based on literature and the likelihood to influence the outcome of interest.

### Statistical analysis

A descriptive analysis of sociodemographic characteristics of the participants was carried out. The proportion of exclusive breastfeeding among the study participants was estimated as: (Number of infants born between 1 January 2011 and 31 December 2013 who were exclusively breastfed / Total number of infants born between 1 January 2011 and 31 December 2013) × 100. The association between the dependent and the independent variables was explored using logistic regression model. The independent variables that were significant at *p* <  0.05 in the crude logistic regression model were entered together into an adjusted model. Data analysis was done using Stata version 14.2 and the results was presented in tables with summary statistics at 95% confidence intervals (CI).

## Results

### Sociodemographic information

Table 1 presents the sociodemographic information of study participants. The mean age was 27.9 years (SD = 7.2). The majority of the study participants (72.78%) were of the Ga-Dangme ethnic group and a large proportion (91.2%) were of the Christian faith. More than half (61.0%) of the participants’ households were headed by males with an average household size was 6.5. A large percentage (71.2%) of study participants delivered in a health facility. More than half (64.0%) of the study participants initiated antenatal clinic visits in the second trimester of their last pregnancy. The proportion of mothers who exclusively breastfed for 6 months was 71.0%.

**Table 1:** Sociodemographic characteristics of the study participants

Characteristics	Frequency^a^	Proportion (%)
Age group (in years)		
< 20	231	12.4
20–24	424	22.7
25–29	483	25.8
30 +	732	39.1
Mean = 27.89 (sd = 7.15)		
Ethnicity		
Ga-Dangme	1361	72.8
Akan	102	5.5
Ewe	287	15.4
Mole- Dagbani	106	5.7
Other tribes	14	0.8
Religion		
Christianity	1706	91.2
Islam	119	6.4
Traditional	28	1.5
Other religions	17	0.9
Occupation		
Unemployed	388	20.8
Farmer	307	16.4
Artisan	219	11.7
Trader	594	31.8
Civil servant	33	1.8
Student	289	15.5
Others	40	2.1
Level of education		
No education	693	37.1
Primary	284	15.2
Junior high school level	656	35.1
Senior high school level and above	237	12.7
Marital status		
Single	543	29.5
Married	382	20.7
Separated/divorced	36	1.9
Cohabiting	882	47.9
Sex of household head		
Female	730	39.0
Male	1140	61.0
Household size		
Less than six	956	51.1
More than five	914	48.9
Mean = 6.45 (SD = 4.33)		
District of residence		
Shai-Osudoku	836	44.7
Ningo-Prampram	1034	55.3
Parity		
Parity 1	520	27.8
Parity 2	429	22.9
Parity 3	374	20.0
Parity3+	547	29.
Delivery place		
Health facility	1332	71.23
Outside health facility	538	28.8
Timing of ANC initiation		
First trimester	525	28.1
Second trimester	1196	64.0
Third trimester	147	7.9
Babies age at time of interview	0.5–1.6 years	
Breastfeeding type		
Not exclusive	543	29.0
Exclusive	1327	71.0
Exclusive breastfeeding rate	**71.0%**	

*n* = 1870; *SD* standard deviation ^a^ number of respondents across some categories may not add up to 1870 due to missing data

### Unadjusted and adjusted odds ratio of determinants of exclusive breastfeeding

Table 2 presents the unadjusted and adjusted Odds Ratio (OR) at 95% CI of socioeconomic and demographic determinants of 6 months exclusive breastfeeding in the Dodowa Health and Demographic Surveillance site. In the unadjusted model, maternal age, occupation, place of delivery, parity, household size and sex of household head had statistically significant associations with 6 months exclusive breastfeeding.

**Table 2:** Unadjusted and adjusted odds ratios of factors associated with 6 months exclusive breastfeeding

Characteristics	Crude		Adjusted^b^	
Age group (in years)	COR (95% CI)	*P* - values	AOR (95% CI)	*P* - values
< 20	1.00		1.00	
20–24	1.24 (0.89, 1.74)	0.205	1.46 (0.98, 2.15)	0.060
25–29	1.53 (1.10, 2.13)^a^	0.012	1.93 (1.25, 2.99)^a^	0.003
30 +	1.75 (1.28, 2.40)^a^	< 0.001	1.91 (1.91, 3.08)^a^	0.007
Marital status				
Single	1.00			
Married	1.22 (0.91, 1.63)	0.193		
Separated/divorced	0.83 (0.40, 1.70)	0.606		
Cohabiting	0.94 (0.74, 1.19)	0.595		
Education				
No education	1.00			
Primary	0.81 (0.62, 1.05)	0.110		
Junior school level	1.03 (0.79, 1.34)	0.844		
SHS and above	1.08 (0.73, 1.61)	0.694		
Occupation				
Unemployed	1.00		1.00	
Farmer	1.77 (1.23, 2.55)^a^	0.002	1.24 (0.82, 1.87)	0.296
Artisan	0.70 (0.49, 1.00)^a^	0.049	0.64 (0.43, 0.96)^a^	0.032
Trader	0.91 (0.69, 1.21)	0.512	1.04 (0.76, 1.43)	0.805
Civil servant	0.91 (0.42, 1.97)	0.812	0.66 (0.28, 1.58)	0.350
Student	0.82 (0.59, 1.14)	0.242	1.04 (0.71, 1.53)	0.838
Others	0.73 (0.37, 1.46)	0.379	0.89 (0.42, 1.85)	0.750
Delivery place				
Health facility	1.00		1.00	
Outside health facility	1.51 (1.20, 1.90)^a^	< 0.001	1.13 (0.87, 1.46)	0.367
Parity				
Parity 1	1.00		1.00	
Parity 2	0.91 (0.79, 1.36)	0.793	0.91 (0.66, 1.26)	0.593
Parity 3	1.17 (0.88, 1.56)	0.285	0.95 (0.66, 1.38)	0.802
Parity 3+	1.56 (1.19, 2.04)^a^	0.001	1.12 (0.75, 1.66)	0.585
Household size				
Less than six	1.00		1.00	
More than five	1.33 (1.09, 1.63)^a^	0.005	1.45 (1.16, 1.81)^a^	0.001
Socio-economic status				
Poorest	1.00			
Poorer	1.04 (0.76, 1.43)	0.809		
Poor	1.09 (0.79, 1.49)	0.611		
Less poor	1.02 (0.75, 1.40)	0.891		
Least poor	0.95 (0.69, 1.30)	0.749		
Timing of ANC				
First trimester	1.00			
Second trimester	1.16 (0.93, 1.45)	0.184		
Third trimester	1.27 (0.84, 1.91)	0.254		
Sex of household head				
Female	1.00		1.00	
Male	1.44 (1.18, 1.77)^a^	< 0.001	1.19 (0.95, 1.49)	0.124
District of residence				
Shai-Osudoku	1.00		1.00	
Ningo-Prampram	1.16 (0.12, 0.21)^a^	< 0.001	0.15 (0.1, 0.20)^a^	< 0.001

*CI* confidence interval, *COR* crude odd ratio, *AOR* adjusted odd ratio, ^a^statistically significant. ^b^ Correct classification rate of the model = 72.8%

Women aged 25–29 and 30 + years were 53 and 75% more likely to practice exclusive breastfeeding respectively compared to those aged < 20 years (COR 1.53, 95% CI 1.10, 2.13, COR 1.75, 95% CI 1.28, 2.40). This was statistically significant.

A similar pattern was observed in the adjusted model such that in the presence of other explanatory variables, the odds of mothers practicing 6 months exclusive breastfeeding increased with increasing maternal age. The odds of mothers aged 25–29 and 30 + years were 93 and 91% respectively more likely to practice 6 months exclusive breastfeeding compared to those aged < 20 years (AOR 1.93, 95% CI 1.25, 2.99, AOR 1.91, 95% CI 1.91, 3.08). This was statistically significant.

There was a statistically significant association between occupation and women practicing 6 months exclusive breastfeeding. The odds of a farmer practicing exclusive breastfeeding was 77% more likely compared to those unemployed (COR 1.77, 95% CI 1.23, 2.55).

In the adjusted model, the odds of female artisans to practice 6 months exclusive breastfeeding was 36% less likely compared to those unemployed (AOR 0.64, 95% CI 0.43, 0.96).

In the unadjusted model, the odds of women who delivered outside health facility to practice 6 months exclusive breastfeeding was 51% more likely compared to those who delivered in a health facility (COR 1.51, 95% CI 1.20, 190). This association was not statistically significant (AOR 1.13, 95% CI 0.87, 1.46) in the adjusted model.

In the crude model, the odds of women with parities two to practice 6 months exclusive breastfeeding compared to those with parity one was 91% (COR 0.91, 95% CI 0.79, 1.36). There was an increased odd of 17 and 56% of mothers with parities three and more (OR 1.17, 95% CI 0.88, 1.56, OR 1.56, 95% CI 1.19, 2.04). In the presence of other variables (age, occupation, place of delivery, household size, sex of household head and district of residence), while there was reduced odds of 91 and 95% of women with parities two and three respectively to practice exclusive breastfeeding (AOR 0.91, 95% CI 0.66, 1.26, AOR 0.95, 95% CI 0.66, 1.38), there was increased odds of 12% for mothers with parity more than three (AOR 1.12, 95% CI 0.75, 1.66) in the adjusted model.

There is an increased odds of 33 and 45% in the crude and adjusted model respective of mothers with household size more than five members to practice 6 months exclusive breastfeeding compared to those with household size of less than six (COR 1.33, 95% CI 1.09, 1.63, AOR 1.45, 95% CI 1.16, 1.81). This was also statistically significant.

In the crude analysis, participants who belong to poorer, poor and less poor socio-economic categories were 4, 9 and 2% respectively more likely to practice 6 months exclusive breastfeeding compared to those in poorest category.

There was increased odds of 16 and 27% for participants who initiated antenatal visit in the second and third trimesters respectively to practice 6 months exclusive breastfeeding compared to those who initiated theirs in the first trimester (COR 1.16, 95% CI 0.93, 1.45, COR 1.27, 95% CI 0.84, 1.91). The crude analysis showed a significant association of women whose households were headed by males being 44% more likely to practice 6 months exclusive breastfeeding compared to those in female headed households (COR 1.44, 95% CI 1.18, 1.77).

The district of residence of participant was statistically significantly associated with practicing 6 months exclusive breastfeeding; there was an increased odds of 16% for women from Ningo-Prampram district compared to those from Shai-Osudoku district in the unadjusted analysis (COR 1.16, 95% CI 0.12, 0.21). The odds ratio was reduced in the adjusted model to 0.15 (AOR 0.15, 95% CI 0.12, 0.20). This was statistically significant.

## Discussion

The aim of this study was to estimate the rate and examine socio-economic and demographic determinants of exclusive breastfeeding in two rural districts (Ningo-Prampram and Shai-Osudoku Districts) of Southern Ghana.

The exclusive breastfeeding rate among the study participants in this study was 71.0%. This rate was higher than 52% (24-h period exclusive breastfeeding) reported by 2014 GDHS [25] but lower than 84.3% (6 months exclusive breastfeeding) report by another Ghanaian study [31].

Maternal age, type of occupation, household size and district of residence appear to be strong determinants of exclusive breastfeeding practices after adjusting for other variables.

Older women were more likely to practice exclusive breastfeeding compared to younger (< 20 years) mothers. This result was consistent with studies in other settings which showed that, younger mothers are at an increased risk of early cessation of exclusive breastfeeding [32–36]. Our findings were also similar to a study conducted in Tanzania where participants who were younger were reported to be less likely to initiate breastfeeding within 1 h after child delivery [37].

Although maternal level of education and socio-economic status were found elsewhere to be significantly associated with 24-h recall breastfeeding practices in other studies [38], the current study had not found a statistically significant association between maternal education, socio-economic status and exclusive breastfeeding.

This supported the finding of an earlier study [31] which suggested that educational attainment of mothers was not associated with 6 months exclusive breastfeeding. Nonetheless, the finding of this current study contradicted the results of 6 months exclusive breastfeeding studies in Nigeria [39, 40], Tanzania [37], India [41, 42] and in Ghana [24] which reported that the level of educational attainment of mothers was positively associated with exclusive breastfeeding practice. Perhaps due to the education provided to pregnant women at the well attended antenatal service (87%) [25], the issue of exclusive breastfeeding has become a universal knowledge hence it is not preserved for only educated women in the study area.

The high rate of exclusive breastfeeding among the study participants could also be attributed to education from Reproductive and Child Health (RCH) Centers of the Ghana Health Service in the study area where pregnant women receive antenatal care services with education on breastfeeding practices as shown in another study [31].

The findings showed that, mothers who were self-employed artisans were more likely to practice exclusive breastfeeding. This finding was in line with earlier studies that found self-employed mothers to be more likely to practice 6 months exclusive breastfeeding [43]. This study found that mothers whose households were headed by males were more likely to practice exclusive breastfeeding but this relationship was not statistically significant after adjusting for other explanatory variables. This result was similar to the findings of other studies in Ghana [43] and Malawi [44] where the decision of 6 months and 24 to 26 h exclusive breastfeeding was influenced by spouses and family members respectively.

Mothers with a family size of four and less were more likely to practice exclusive breastfeeding as compared to those with family size above four.

It was also very intriguing to find that the district of residence was significantly associated with exclusive breastfeeding practice among the study participants. Women from Ningo-Prampram District were 15% less likely to practice exclusive breastfeeding compared to those from Shai-Osudoku District. This could be ascribed to the introduction of pregnancy schools in health facilities in Shai-Osudoku District where expectant mothers received education, including how to look after themselves and their babies and the importance of exclusive breastfeeding. The effective RCH in Shai-Osudoku District where pregnant women receive antenatal care services with education on breastfeeding practices could be another contributing factor to the high likelihood of exclusive breastfeeding practice in the Shai-Osudoku District.

### Strength and limitations of the study

Despite the advantage of a large sample and use of population-based data, this study has a number of limitations. First, the outcome was measured based on self-report; recall bias may have influenced underestimation or overestimation of the association between the outcome of interest and the explanation variables. Social desirability bias could also be a limitation to the study as some women might have withheld what they thought to be negative aspects of their breastfeeding practices which may lead to over estimating the proportion of women who exclusively breastfed for 6 months. The study did not explore other factors such as knowledge, initiation, duration and cultural determinants of exclusive breastfeeding which might have some influence on the outcome of interest. This was primarily due to the limited information in the secondary data used. The study was also limited to only two districts in the Greater Accra Region of Ghana hence, limits the generalizability of the findings.

## Conclusions

The majority of the study participants practiced exclusive breastfeeding. Maternal age, type of occupation, household size and district of residence were strong determinants of exclusive breastfeeding practices.

Maintaining access to information on appropriate breastfeeding practices and promotion of exclusive breastfeeding especially among young mothers in the study area is highly recommended. To further understand other factors influencing the practice of exclusive breastfeeding and to design a suitable evidence-based intervention targeting young mothers, we recommend further qualitative study in this area.

## Data Availability

All relevant data supporting the conclusions of this article are included within the article. Any additional information is available from the corresponding author on reasonable request.

## Abbreviations

ANC: Antenatal Care
CI: Confidence Interval
DHDSS: Dodowa Health and Demographic Surveillance System
DHRC: Dodowa Health Research Centre
GDHS: Ghana Demographic Health Survey
JHS: Junior High School
OR: Odd Ratio
PCA: Principal Component Analysis
RCH: Reproductive and Child Health
WHO: World Health Organization
